# Novel Thermophilic Bacterial Laccase for the Degradation of Aromatic Organic Pollutants

**DOI:** 10.3389/fchem.2021.711345

**Published:** 2021-10-20

**Authors:** Nabangshu Sharma, Ivanhoe K.H. Leung

**Affiliations:** ^1^ School of Chemical Sciences, The University of Auckland, Auckland, New Zealand; ^2^ Centre for Green Chemical Science, The University of Auckland, Auckland, New Zealand; ^3^ School of Chemistry, The University of Melbourne, Parkville, VIC, Australia; ^4^ Bio21 Molecular Science and Biotechnology Institute, The University of Melbourne, Parkville, VIC, Australia

**Keywords:** laccase, organic pollutants, enzyme technology, bioremediation, biocatalysts

## Abstract

We identified a putative laccase from the thermophilic bacterium *Geobacillus yumthangensis*. The putative laccase was produced recombinantly and its ability to catalyse the degradation of aromatic organic pollutants was investigated. The putative laccase exhibits broad pH and temperature stability, and, notably, it could catalyse the degradation of organic dyes as well as toxic pollutants including bisphenol A, guaiacol and phenol with a redox mediator. Our work further demonstrates the potential of using oxidative enzymes to break down toxic chemicals that possess major threats to human health and the environment.

## Introduction

Laccases are versatile multi-copper oxidases that catalyse the single-electron oxidation of phenolic and aniline substrates (Su et al., 2018; Arregui et al., 2019). This group of enzymes are found in plants, fungi, insects and bacteria, where they are involved in a range of important biological functions (Alcalde, 2007; Arregui et al., 2019). As laccases possess broad substrate specificity (McLarin and Leung, 2020), their applications as biocatalysts have attracted significant interest in the last 2 decades (Riva, 2006; Rodríguez Couto and Toca Herrera, 2006; Alcalde, 2007; Mate and Alcalde, 2016). For example, laccases have been investigated and applied as biocatalysts to catalyse the conversion of lignin biomass (Liu et al., 2020) as well as the degradation of hazardous contaminants (Bilal et al., 2019). The use of laccases in the food and wine industries has also been explored (Minussi et al., 2007; Osma et al., 2010). Recently, the application of laccases as biosensors to detect phenolic pollutants has also been proposed (Rodríguez-Delgado et al., 2015).

Persistent and emerging organic pollutants are toxic chemicals that are present in the environment including waterways and wastewater effluents (Wania and MacKay, 1996; El-Shahawi et al., 2010; Deblonde et al., 2011; Lapworth et al., 2012). A significant portion of these pollutants are aromatic organic compounds, including but are not limited to pharmaceuticals, endocrine disruptors, and industrial chemicals such as dyes, phthalates and bisphenols. These compounds possess a major threat to human health and the aquatic ecosystem (Eljarrat and Barceló, 2003). The use of enzyme technology as a sustainable means for the degradation of water pollutants has gained considerable momentum in recent years (Sharma et al., 2018). In particular, oxidative enzymes such as laccases have emerged to be promising candidates for wastewater treatments (Gasser, 2014; Unuofin et al., 2019).

We are interested in the studies of bacterial laccases as their production and purification could be readily scaled up (Chauhan et al., 2017) when compared to laccases from other sources (such as fungi), which may require additional engineering steps to facilitate their production and secretion (De Salas et al., 2019; Aza et al., 2021). Although a number of bacterial laccases have been characterised to date (Sharma et al., 2007; Chandra and Chowdhary, 2015; Chauhan et al., 2017; Sharma and Leung, 2021), we believe there is still a need to expand the pool of bacterial laccases that could be used for pollutant degradation. This is because the employment of laccases for wastewater treatments is challenging. For example, wastewater discharged from different sources may have drastically different physical characteristics (such as pH and impurities) (Muttamara, 1996). Hence, having a library of laccases that could work under different operational conditions would facilitate their use. Thermophilic bacteria are good sources of enzyme biocatalysts as they are typically more thermostable than enzymes from mesophilic bacteria (Atalah et al., 2019). *Geobacillus yumthangensis* is a thermophilic bacterium that has recently been isolated from a hot spring in North East India (Najar et al., 2018a; Najar et al., 2018b), hence its genome may encode novel laccases that have not been characterised before. Herein, we report our work in the identification of a putative laccase from *G. yumthangensis* (pLac_*Gy*_) by using bioinformatics. pLac_*Gy*_ was produced recombinantly by using the *Escherichia coli* expression system. Following the purification of pLac_*Gy*_, its pH, temperature and solvent tolerance profiles were measured. By using organic dyes and phenolic pollutants as model compounds, the ability of pLac_*Gy*_ to degrade aromatic organic compounds was also explored.

## Materials and Methods

### Materials

All chemicals were purchased from Abcam, AK Scientific, Bio-Rad, Merck, Thermo Fisher Scientific and Sigma-Aldrich unless otherwise stated. Plasmid [pET-28a(+)] encoding full length pLac_*Gy*_ was purchased from GenScript (Supplementary Table S1).

### Sequence Alignment Search

The National Center for Biotechnology Information (NCBI) Basic Local Alignment Search Tool (BLAST) was used for the protein sequence alignment search. The input sequence was Lac1326 from an uncultured bacterium (NCBI accession code: AKN79754) (Yang et al., 2018).

### Production of Recombinant pLac_*Gy*_

Plasmid encoding pLac_*Gy*_ was transformed into *E. coli* BL21 (DE3) competent cells (Sigma-Aldrich) and plated on an LB agar plate containing 50 µg/ml kanamycin. A single colony was used to inoculate 100 ml of 2-YT medium supplemented with 50 µg/ml kanamycin, which was grown in a shaking incubator at 37°C overnight. The starter culture was then diluted (in a 1:100 ratio) with fresh 2-YT medium supplemented with 50 µg/ml kanamycin and 0.25 mM CuCl_2_, which was then incubated at 37°C with shaking until it reached OD_600_ of 0.6, at which point protein expression was induced using 0.1 mM isopropyl 1-thio-β-D-galactopyranoside (final concentration). After induction, the culture was further incubated at 18°C with shaking for 16 h. Bacterial cells were harvested by centrifugation. The harvested cells were stored at −80°C until use.

### Purification of Recombinant pLac_*Gy*_

Two purification methods, one using heat precipitation and the other using column chromatography, were investigated.

Heat precipitation method: The harvested cells were first resuspended in buffer A (50 mM Tris-HCl, pH 7.5, 150 mM NaCl, 5% glycerol) and then lysed by sonication. After removal of cell debris by centrifugation, the cell lysate was incubated at different temperatures (between 30 and 80°C) for 20 min to investigate the optimal condition for *E. coli* protein denaturation and precipitation. This was followed by cooling the sample on ice for 10 min. After removal of the precipitated proteins by centrifugation, the supernatant was analysed by sodium dodecyl sulphate-polyacrylamide gel electrophoresis (SDS-PAGE) to examine the presence and purity of pLac_*Gy*_. We were unable to fully separate pLac_*Gy*_ from other thermostable *E. coli* proteins by using this method (see Results and discussion for further information).

Column chromatography method: The harvested cells were first resuspended in buffer A and then lysed by sonication. After removal of cell debris by centrifugation, the cell lysate was loaded onto a His GraviTrap column charged with Ni^2+^ ions. The polyhistidine-tagged protein was eluted using buffer A supplemented with 500 mM imidazole. Protein purity was assessed using SDS-PAGE. The purified pLac_*Gy*_ was buffer exchanged with buffer A to remove the imidazole and concentrated by spin concentration. Highly purified pLac_*Gy*_ (∼95% as judged by SDS-PAGE) was obtained using this method.

### Tandem Mass Spectrometry for Protein Sequencing

In-gel trypsin digestion, and tandem mass spectrometry measurement and analysis of the gel band-of-interest were performed as described in Li et al., 2019.

### Differential Scanning Fluorimetry

Differential scanning fluorimetry assay was carried out by using a QuantStudio 3 Real-Time PCR system (Thermo Fisher Scientific). The assay was carried out using 20 μM pLac_*Gy*_ in Britton-Robinson buffer (pH 5.0). Protein unfolding was monitored by measuring the fluorescence of the SYPRO Orange dye. The dye stock (5,000× concentrate) was first diluted in Britton-Robinson buffer (pH 5.0) to a 200× concentrate before diluting by 5 times into the sample. Temperature was increased from 25 to 95°C at 1°C increment every 60 s. Experiment was performed in triplicate. For determination of protein melting temperature, the fluorescence of the dye was plotted against temperature on GraphPad Prism 5.0 using the Boltzmann sigmoidal function.

### Laccase Activity Assay

The 2,2′-azino-bis(3-ethylbenzothiazoline-6-sulfonic acid) (ABTS) assay was used to monitor laccase activity (Johannes and Majcherczyk, 2000). Assay mixture included 0.5 µM pLac_*Gy*_, 1 mM ABTS (unless otherwise stated), 50 µM CuCl_2_ in 0.04 M Britton-Robinson buffer, pH 5.0 (unless otherwise stated). The volume was 100 µL. Unless otherwise stated, reaction mixture was incubated at 60°C with shaking at 500 rpm for 20 min. The oxidation of ABTS to ABTS^·+^ was monitored spectrophotometrically by following the increase of absorbance at 420 nm. All assays were performed in triplicates.

Specific activity of the laccase was calculated by the following equation:

$$\mathrm{Specific activity}=\left[\Delta \mathrm{Abs}\times V_{T}\times 1000\right]/\left[\varepsilon \times d\times V_{E}\times t\times C_{E}\right]$$

In which ΔAbs = changes of absorbance at 420 nm, V_T_ = volume of reaction mixture (in mL), ε = molar extinction coefficient of ABTS^·+^ at 420 nm (in M^−1^ cm^−1^), d = path length of cuvette/well (in cm), V_E_ = volume of stock enzyme solution used (in mL), t = reaction time (in min), C_E_ = concentration of stock enzyme solution (in mg/mL). The unit of specific activity was U/mg, in which one unit (U) of enzyme activity is defined as the amount of enzyme that is needed to catalyse the oxidisation of 1 μmol of ABTS in 1 min.

For the calculation of enzyme kinetics parameters, absorbance was monitored 5 min after the start of the reaction to obtain initial reaction rates. Initial rates were plotted against substrate concentrations and the data was fitted by non-linear regression to the Michaelis-Menten equation (shown below) with SigmaPlot 14.0 (Systat Software, United States) to obtain the kinetic parameters.

$$v=\frac{V_{\max}\left[S\right]}{K_{m}+\left[S\right]}\;$$

In which v = initial reaction rates, V_max_ = maximum reaction rate, K_m_ = Michaelis constant and [S] = substrate concentration, which can be approximated as [S]_total_ at the start of the reaction.

### Laccase Stability Assay by Residual Enzyme Activity

10 μM pLac_*Gy*_ was incubated in the test condition (e.g. different pH, temperature, organic solvent/aqueous mixture) for 6 h. The solution was then centrifuged at 12,000 rpm for 10 min to remove protein debris. The supernatant was diluted 20 times in Britton-Robinson buffer (pH 5.0) and the residual activity was measured at 60°C in presence of 1 mM ABTS and 50 μM CuCl_2_. The activity of the enzyme without incubation measured at the optimal pH and temperature (pH 5.0 at 60°C) was set at 100%.

### Measurement of Organic Dye Decolourisation

Reaction mixture included 1 µM pLac_*Gy*_, 1 mM ABTS, 50 µM CuCl_2_, 0.05 mg/ml organic dye in Britton-Robinson buffer (pH 5.0). The mixture was incubated overnight at 60°C with shaking at 500 rpm. Dye decolourisation was determined by measuring the decrease in absorbance using the following wavelengths for the different dyes: alizarin (496 nm), acid red 27 (520 nm), Congo red (490 nm), bromophenol blue (592 nm), Coomassie brilliant blue R-250 (556 nm), malachite green (624 nm) and indigo carmine (610 nm). Dye decolourisation was expressed in terms of % decolourisation.

$$\%\mathrm{Decolorisation}=\frac{\mathrm{Initial absorbance}-\mathrm{Final absorbance}}{\mathrm{Initial absorbance}}\times 100\%$$

### Determination of the Removal of Phenolic Pollutants

Reaction mixture included 1 µM pLac_*Gy*_, 1 mM ABTS, 50 µM CuCl_2_, 0.5 mM phenol, guaiacol or bisphenol A (BPA), in Britton-Robinson buffer (pH 5.0). The volume was 1 ml. The mixture was incubated overnight at 60°C with shaking at 500 rpm. To estimate the removal of the phenolic compounds, 300 µL of the reaction mixture was added to a solution containing 10 μL 4-aminoantipyrine (4-AAP, 0.1 M), 10 μL potassium ferricyanide solution (0.2 M) and 700 μL of Britton-Robinson buffer (pH 9.0). The mixture was then incubated at 25°C with shaking at 100 rpm for 10 min. The absorbance of the reaction mixture was then measured at 505 nm and the removal efficiency was determined by the following equation.

$$\%\mathrm{Removal}=\frac{\mathrm{Initial absorbance}-\mathrm{Final absorbance}}{\mathrm{Initial absorbance}}\times 100\%$$

## Results and Discussion

### Identification of pLac_*Gy*_ Through Sequence Alignment Search

**FIGURE 1:**
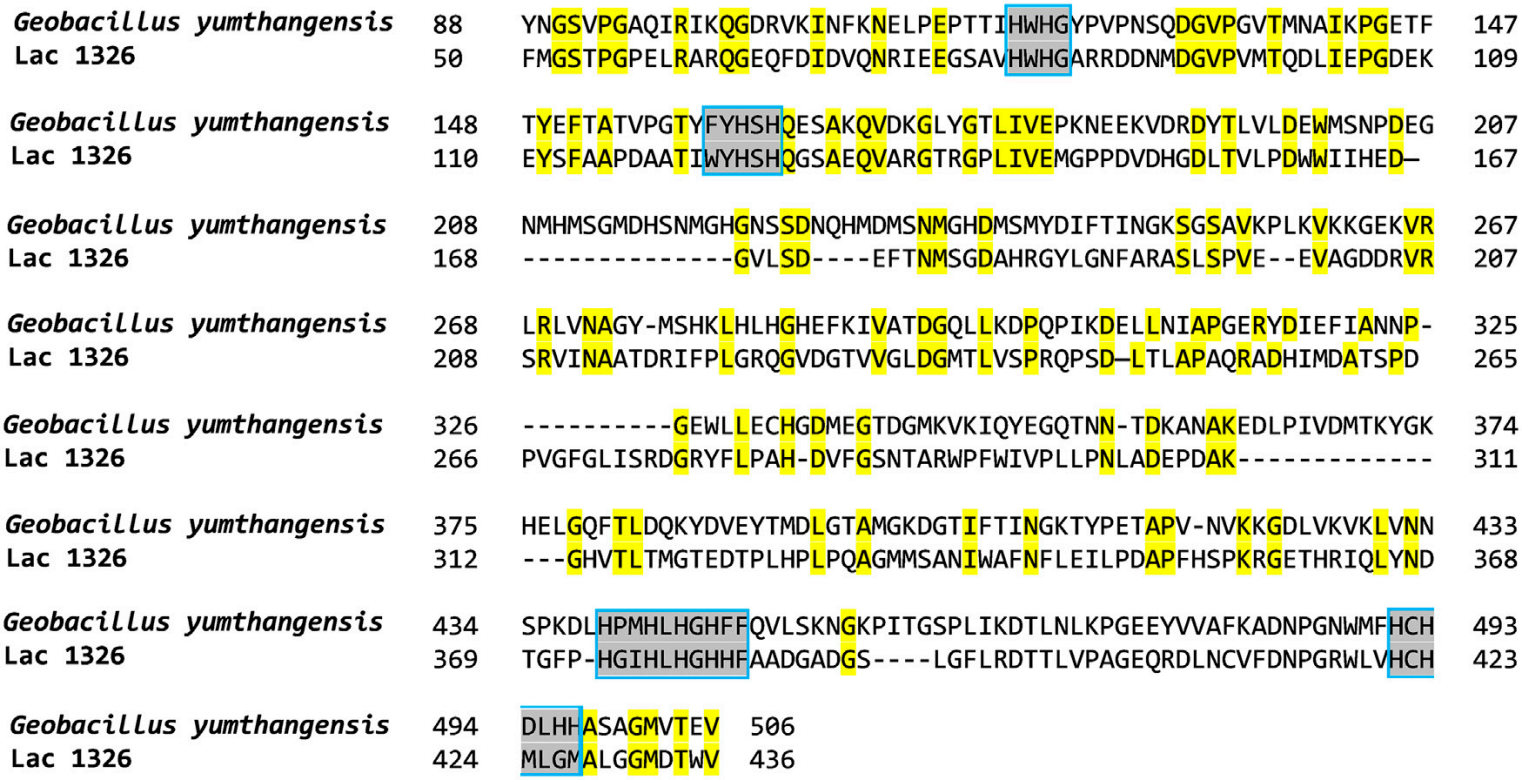
Amino acid sequence alignment of pLac_*Gy*_ and Lac1326. Conserved residues are highlighted in yellow. The four highly conserved amino acid sequences found in laccases are marked by a box and highlighted in grey.

In order to hunt for novel laccases that are thermostable, we set out to study thermophilic bacteria that have recently been isolated and whose genome has been sequenced. In this study, we focus on *G. yumthangensis*, which is a thermophilic bacterium isolated from a hot spring in the north-eastern Indian state of Sikkim in 2018 (NCBI accession code: NWUZ01000001) (Najar et al., 2018a; Najar et al., 2018b). Our approach to identify novel laccases from *G. yumthangensis* was by protein sequence similarity searches. This is because most laccases that have been characterised to date contain four highly conserved amino acid sequence regions (for the coordination to copper ions and for substrate binding at the active site) (Kumar et al., 2003). These conserved sequences therefore form the basis of our search. We decided to use Lac1326, a recently reported dye-degrading laccase isolated from an uncultured bacterium extracted from the South China Sea, as the query sequence (NCBI accession code: AKN79754) (Yang et al., 2018). By using the NCBI BLAST search function, we obtained a putative protein-of-interest (pLac_*Gy*_) from *G. yumthangensis* that contains ∼30% homology with Lac1326 as well as containing the four conserved regions that are commonly found in laccases (NCBI accession code: PDM40740) (Figure 1). Phylogenetic analysis of pLac_*Gy*_ with 24 other related bacterial laccases showed that it has high sequence homology (>90%) with putative laccases from other Geobacillus and Parageobacillus species. It was also closely related to a laccase from *Bacillus methanolicus* (Supplementary Figure S1).

### Production, Purification and Characterisation of Recombinant pLac_*Gy*_

Upon the identification of a putative laccase through sequence similarity searches, we proceeded to produce pLac_*Gy*_ recombinantly so that we could test its laccase activity. Recombinant pLac_*Gy*_ was produced as a soluble enzyme by using *E. coli* as an expression host. The enzyme could be readily extracted by sonication. SDS-PAGE analysis of the cell lysate showed a thick intense band at ∼60 kDa (Supplementary Figure S2a), which matches the predicted molecular weight of 58.6 kDa plus the molecular weight of the N-terminal polyhistidine tag with a thrombin cleavage site (2.18 kDa). The gel band was further analysed by tandem mass spectrometry, which confirmed the identity of the overexpressed protein as pLac_*Gy*_.

We then optimised the purification of pLac_*Gy*_ so that highly purified enzyme could be obtained for further downstream studies. Two methods were tested. The first purification method was heat precipitation, a strategy that is commonly employed to purify thermophilic enzymes expressed in mesophilic hosts (Kirk and Cowan, 1995). We first investigated the temperature that is needed to precipitate the host *E. coli* proteins from the cell lysate (30–80°C). We found that it was possible to obtain relatively pure pLac_*Gy*_ (∼90% purity) by heating the cell lysate at 70°C (Supplementary Figure S2a). To investigate whether the heat-purified pLac_*Gy*_ possesses laccase activity, we tested its ability to catalyse the oxidation of ABTS, which is a commonly used substrate for laccases (Johannes and Majcherczyk, 2000). We found that, upon the addition of the heat-purified pLac_*Gy*_, the ABTS solution turned from colourless to green, indicating the formation of ABTS^·+^, which is a highly stable cation radical that absorbs strongly at 420 nm (ε_420 nm_ = 36,000 M^−1^ cm^−1^) (Collins et al., 1998). We found that the heat-purified pLac_*Gy*_ supernatant retained ∼90% of laccase activity when compared to the same cell lysate without any heat treatments (Supplementary Figure S2b). However, heating the cell lysate beyond 70°C caused a significant decrease in laccase activity. As control, heat treatment (at 70°C) was also performed with a cell lysate sample that does not contain pLac_*Gy*_ (i.e., *E. coli* cells grown without the plasmid encoding pLac_*Gy*_). Surprisingly, we also observed laccase activity in the control sample (although the activity was less than those observed from the cell lysate overexpressed with pLac_*Gy*_). This could be due to the presence of native *E. coli* laccases or other *E. coli* enzymes that are capable of catalysing the oxidation of ABTS. Tandem mass spectrometric analysis of the heat-treated *E. coli* cell lysate (that did not contain pLac_*Gy*_) indicated the presence of an enzyme CueO, which has previously been reported to exhibit laccase activity (Ma et al., 2017). Thus, we concluded that the heat precipitation purification method is not suitable for the purification of pLac_*Gy*_ from *E. coli* as the presence of native *E. coli* enzymes that possess laccase activity may affect further downstream characterisation of pLac_*Gy*_.

Following the results from the heat precipitation method, a second purification method employing column chromatography was investigated. We decided to use immobilised metal affinity chromatography (IMAC) to purify the recombinantly produced pLac_*Gy*_ since it was expressed with an N-terminal polyhistidine tag. We found that this method is highly efficient, and we were able to obtain pLac_*Gy*_ with more than 95% purity after only one purification step (Supplementary Figure S3). In-gel digestion and tandem mass spectrometry analyses were also conducted to confirm that there were no native *E. coli* laccases or oxidative enzymes present in the purified sample. The typical yield of pure pLac_*Gy*_ using the IMAC purification method was found to be ∼3 mg per litre of culture media. We then tested the IMAC-purified pLac_*Gy*_ by the ABTS assay. Surprisingly, we found that the IMAC-purified pLac_*Gy*_ possess little laccase activity (Supplementary Figure S4). Heterologous expression of laccases in *E. coli* usually results in incomplete copper loading due to the copper homeostasis system of the cell (Rensing and Grass, 2003; Durão et al., 2008). Moreover, as the purification column was immobilised with Ni^2+^ ions, it is possible that the IMAC-purified pLac_*Gy*_ was stripped of its Cu^2+^ ions. We therefore tested whether supplementing the IMAC-purified pLac_*Gy*_ with Cu^2+^ may improve its activity. Indeed, we found that, upon the addition of 50 µM Cu^2+^, the IMAC-purified pLac_*Gy*_ could readily catalyse the oxidation of ABTS with a specific enzyme activity of 49.91 mU/mg (Supplementary Figure S4). However, excess Cu^2+^ (100 µM or more) may lead to the precipitation of the protein (Supplementary Figure S5). Under this optimised condition (IMAC-purified pLac_*Gy*_ supplemented with 50 µM Cu^2+^), the Michaelis constant (K_m_) of the enzyme was found to be 6.35 ± 2.01 mM, and the corresponding values for maximum velocity (V_max_), catalytic constant (k_cat_) and catalytic efficiency (k_cat_/K_m_) are 12.16 ± 2.69 μM min^−1^, 0.41 ± 0.04 s^−1^ and 0.07 ± 0.02 mM^−1^ s^−1^ respectively (Supplementary Figure S6).

### Effect of pH and Temperature on Activity and Stability of pLac_*Gy*_

**FIGURE 2:**
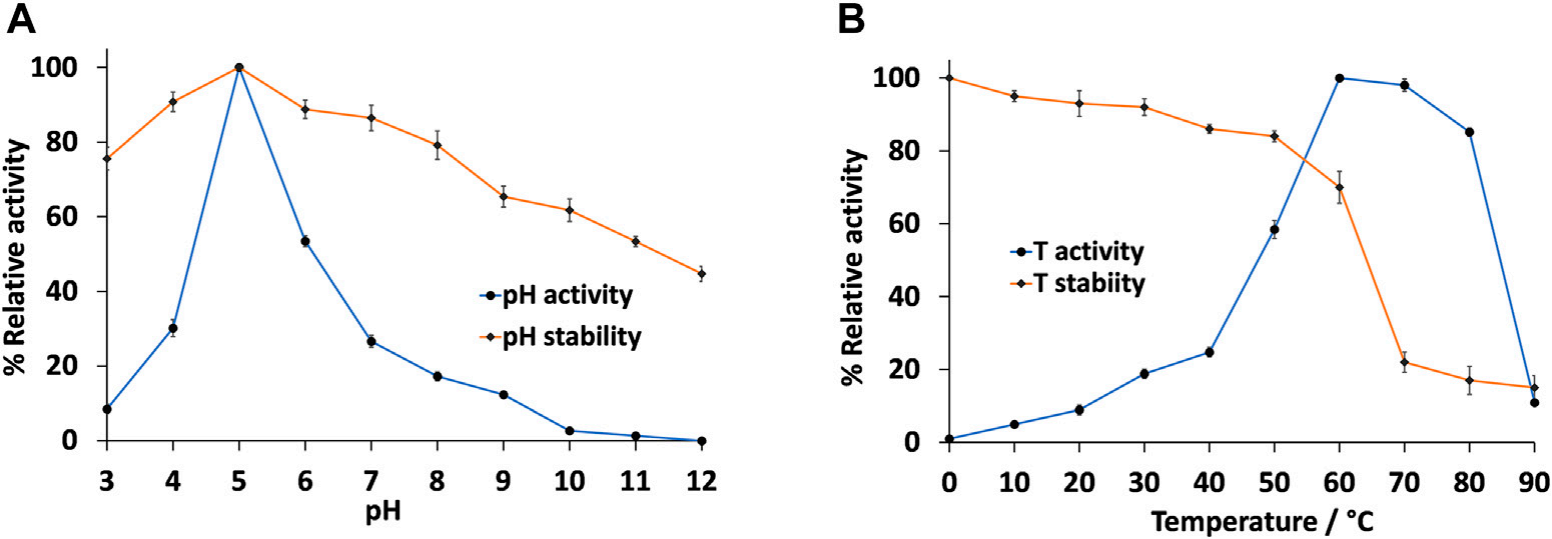
Effect of **(A)** pH and **(B)** temperature on the activity and stability of pLac_*Gy*_. Unless otherwise stated, assay temperature was 60°C. Assay mixture included 0.5 µM pLac_*Gy*_, 1 mM ABTS, 50 µM CuCl_2_ in 0.04 M Britton-Robinson buffer (pH 5.0). For enzyme stability measurements, residual activity was measured at 60°C in presence of 1 mM ABTS and 50 μM CuCl_2_ in 0.04 M Britton-Robinson buffer (pH 5.0) after pre-incubating the enzyme in the test condition for 6 h. The activity of the enzyme without pre-incubation measured at the pH 5.0 at 60°C was set at 100%. All experiments were conducted in triplicates and error bars represent standard deviations.

We then further characterised the IMAC-purified pLac_*Gy*_ by studying the effect of pH and temperature to the activity and stability of the enzyme. We found that pLac_*Gy*_ was most active at slightly acidic pH, with maximum activity observed at pH 5.0 and ∼50% activity at pH 6.0 (Figure 2A). However, its activity dropped drastically on either side of this pH range. The narrow pH range of 5.0–6.0 is in line with many bacterial laccases that have been characterised to date (i.e. they typically function at specific narrow pH ranges) (Arregui et al., 2019). In contrast to its narrow pH activity profile, pLac_*Gy*_ was found to be relatively stable over a wide range of pH (Figure 2A). We found that the enzyme was most stable at pH 5.0. It is also relatively stable towards highly acidic or basic conditions. For example, it retained ∼50% residual activity after incubating at pH 12.0 for 6 h. It also retained ∼75% residual activity after incubating at pH 3.0 for 6 h. The significant stability of pLac_*Gy*_ at extreme pH conditions could be advantageous as a biocatalyst as it may allow less stringent storage and/or operational conditions.

We then investigated the effect of temperature to the activity and stability of pLac_*Gy*_. Our results showed the optimal temperature for pLac_*Gy*_ was 60°C. It retained close to 85% activity at 80°C (Figure 2B). This is not surprising given pLac_*Gy*_ is encoded by a thermophilic bacterium. Similar to many thermophilic enzymes (Akanuma et al., 2019), pLac_*Gy*_ did not show any significant activity in the low temperature ranges. For example, it was five times less active at 40 °C than when it was at 60°C. We also showed that pLac_*Gy*_ is stable at 60 °C. However, its stability dropped beyond 70 °C (Figure 2B). The thermal denaturation temperature of the enzyme was found to be 65°C (Supplementary Figure S7). One of the requirements for a thermostable industrial enzyme biocatalyst is that it should maintain its activity at 60°C (Rigoldi et al., 2018). Hence, pLac_*Gy*_ could be an attractive candidate for biocatalytic operations at elevated temperatures.

### Tolerance of pLac_*Gy*_ Towards Impurities

Wastewater, especially those that come from industrial waste effluents, may contain inorganic contaminants such as salts as well as organic contaminants such as water miscible solvents (Muttamara, 1996). These contaminants may denature proteins and therefore affect the efficacy of enzyme biocatalysts. We therefore studied the tolerance of pLac_*Gy*_ towards such inorganic and organic contaminants. This was conducted by pre-incubating the enzyme in different concentrations of contaminants for 6 h, followed by diluting the enzyme in aqueous buffer to measure its residual activity. We first tested the tolerance of pLac_*Gy*_ to NaCl. Although the presence of a moderate amount of salt (such as NaCl) may improve the solubility of proteins (e.g., 100–150 mM NaCl is typically added in purification buffers for soluble proteins as in the case pLac_*Gy*_), at very high salt concentrations (e.g., >1 M), the high ionic strength may destabilise the proteins, which could lead to protein precipitation (Damodaran and Kinsella, 1981). Our results showed that pLac_*Gy*_ was stable at the NaCl concentration range that we have tested (0.1, 0.5 and 1.0 M) (Table 1), indicating that it can tolerate solutions with moderate to high ionic strengths. We then proceeded to test the stability of pLac_*Gy*_ to different water miscible organic solvents. Five organic solvents, including methanol, ethanol, acetone, acetonitrile and dimethyl sulfoxide (DMSO), were tested at three different concentrations [10, 30 and 50% (v/v)]. However, it was found that pLac_*Gy*_ was unstable in all the tested conditions (Table 1), indicating that these organic solvents may have an adverse effect on the native conformation of the enzyme. These could be due to the ability of organic solvents to disrupt hydrogen bond interactions, which may lead to protein unfolding (Gekko et al., 1998; Rodakiewicz-Nowak et al., 2000). Although laccases with some tolerance for organic solvents have been isolated before (Guan et al., 2014; Gogotya et al., 2021), most enzyme biocatalysts do not survive in organic solvent-aqueous mixture in their native form (Khmelnitsky et al., 1991). Further optimisations through enzyme immobilisation, organic solvent adaptation (Wu et al., 2019) and/or directed evolution are needed to improve the organic solvent tolerance of pLac_*Gy*_.

**TABLE 1:** Effect of organic solvents and NaCl on the stability of pLac_*Gy*_. Residual activity was measured at 60°C in presence of 1 mM ABTS and 50 μM CuCl_2_ in 0.04 M Britton-Robinson buffer (pH 5.0) after pre-incubating the enzyme in the test condition for 6 h. The activity of the enzyme without pre-incubation measured at the pH 5.0 at 60°C was set at 100%. All experiments were conducted in triplicates and uncertainties represent standard deviations.

Buffer/Organic solvent	Concentration (% v/v)	Residual activity (%) (pLac_*Gy*_)
Britton-Robinson buffer	---------	100 ± 0.0
Deionised water	---------	91.9 ± 1.8
Methanol	10	23.8 ± 2.1
—	30	7.1 ± 1.8
—	50	2.9 ± 1.2
Ethanol	10	25.6 ± 2.3
—	30	7.0 ± 2.7
—	50	3.8 ± 1.3
Acetone	10	23.0 ± 2.1
—	30	6.9 ± 1.9
—	50	2.8 ± 1.9
Acetonitrile	10	9.7 ± 1.8
—	30	7.5 ± 1.7
—	50	2.7 ± 1.1
DMSO	10	11.5 ± 1.6
—	30	3.3 ± 1.2
—	50	2.6 ± 1.0

Salt	Concentration (mM)	Residual activity (%) (pLac_*Gy*_)
NaCl	100	76.7 ± 2.8
—	500	75.0 ± 2.7
—	1,000	65.6 ± 3.0

### Degradation of Aromatic Compounds by pLac_*Gy*_

To test the ability of pLac_*Gy*_ to degrade aromatic organic pollutants, we first employed organic dyes as model compounds. Seven organic dyes, including alizarin, acid red 27, Congo red, bromophenol blue, Coomassie brilliant blue R-250, malachite green and indigo carmine (Supplementary Figure S8), were tested. Previous studies showed that laccases could catalyse the oxidation of aromatic organic compounds either directly (e.g., by accepting the aromatic compounds as substrates) or indirectly via redox mediators such as stable radicals, which could act as electron shuttle between the enzyme and compounds (Wells et al., 2006; Morozova et al., 2007). We therefore tested the ability of pLac_*Gy*_ to catalyse the oxidation of these organic dyes with and without a redox mediator (ABTS). As the addition of ABTS may affect the absorbance of the dyes, their absorption profiles with and without ABTS were measured (Supplementary Figures S9–S16). However, such influence was found to be minimal.

**FIGURE 3:**
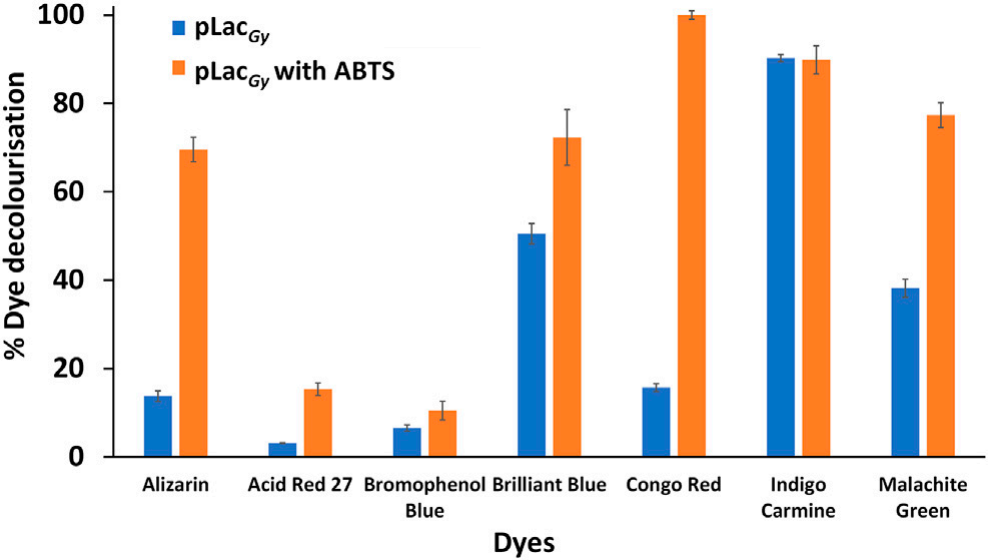
Decolourisation of organic dyes with pLac_*Gy*_ with and without ABTS as redox mediator. Assay mixture included 1 µM pLac_*Gy*_, 1 mM ABTS, 50 µM CuCl_2_, 0.05 mg/ml organic dye in 0.04 M Britton-Robinson buffer (pH 5.0). The mixture was incubated for 18 h at 60°C with shaking at 500 rpm. All experiments were conducted in triplicates and error bars represent standard deviations.

Our results showed that, in the absence of a redox mediator, pLac_*Gy*_ could only catalyse the decolourisation of three (out of the seven tested) dyes. The decolourisation of indigo carmine was the most efficient (90% decolourisation overnight), which is followed by brilliant blue and malachite green (∼40% overnight) (Figure 3). The degradation of the four other dyes was negligible. We then repeated the experiments in the presence of ABTS, which, upon oxidation to ABTS^·+^, could facilitate the oxidation of aromatic organic dyes. We found that the use of ABTS as a redox mediator expanded the range of aromatic organic dyes that could be decolourised. In addition to brilliant blue, indigo carmine and malachite green, we found that ABTS could also mediate the decolourisation of Congo red and alizarin (Figure 3). However, there was still no significant decolourisation of acid red 27 and bromophenol blue. We observed the formation of precipitates during the decolourisation of brilliant blue but no precipitations were observed with the decolourisation of indigo carmine, malachite green, Congo red and alizarin. This is in line with previous studies, which showed that the oxidised dyes may break down into small soluble fragments. In addition, some of these fragments (e.g., quinones and isatin sulfonates) may also react non-enzymatically with each other to form insoluble polymers (Chivukula and Renganathan, 1995; Zucca et al., 2015; Li et al., 2017; Wang et al., 2017). As control, no decolouristion was observed in the absence of pLac_*Gy*_ over the same period.

Comparing the redox potential of the dyes, we found that compounds with relatively low redox potentials (e.g. indigo carmine (E° ∼0.68 V versus normal hydrogen electrode) (Manjunatha, 2018), Coomassie brilliant blue (E° ∼0.60 V) (Devi et al., 2018) and malachite green (E° ∼0.70 V) (Hou et al., 2013)) are direct substrate of pLac_*Gy*_. These dyes have similar redox potential to ABTS (E° = 0.69 V) (Mateljak et al., 2019). In contrast, pLac_*Gy*_ was not able to directly catalyse the oxidation of dyes that possess relatively high redox potentials (e.g. alizarin (E° ∼0.98 V) (Mahanthesha et al., 2009), Congo red (E° ∼0.88–0.91 V) (Shetti et al., 2019), acid red 27 (E° ∼1.26 V) (Zille et al., 2008) and bromophenol blue (E° ∼1.06 V) (Yang et al., 2007)) although the decolourisation of alizarin and Congo red could be achieved by the use of a redox mediator. The inability of pLac_*Gy*_ to directly catalyse the oxidation of dyes that possess a relatively high redox potential is not surprising as most laccases have a redox potential of between 0.4 and 0.8 V at their T1 copper site. The inability of pLac_*Gy*_ to indirectly catalyse the oxidation of acid red 27 and bromophenol blue could be due to steric (e.g. bromophenol blue possesses bulky bromo groups that may block redox mediator to reach the redox labile hydroxyl groups), redox potential or a combination of both effects.

Finally, the application of pLac_*Gy*_ to degrade phenolic pollutants was tested. Phenol, guaiacol and BPA were chosen for this study as these compounds are known environmental contaminants with high toxicity (LD_50_ < 1,000 mg/kg). These compounds were incubated with pLac_*Gy*_ in the absence and presence of ABTS. By using a colorimetric assay that monitors the formation of coloured complexes between phenolic compounds and 4-AAP (Asadgol et al., 2014), we found that, in the presence of ABTS, pLac_*Gy*_ could readily eliminate these phenolic compounds from aqueous solution (Table 2). The removal of guaiacol was most the efficient (93%), which is followed by phenol and BPA (55 and 25% respectively) (Table 2). Minimal degradation of the phenolic compounds was observed in the absence of ABTS or in the absence of pLac_*Gy*_. Our results therefore demonstrate the potential of pLac_*Gy*_ to degrade aromatic pollutants in aqueous solution.

**TABLE 2:** Ability of pLac_*Gy*_ to remove phenolic pollutants in absence and presence of ABTS as a redox mediator. Assay mixture included 1 µM pLac_*Gy*_, 1 mM ABTS, 50 µM CuCl_2_, 0.5 mM phenolic compound in 0.04 M Britton-Robinson buffer (pH 5.0). The mixture was incubated overnight at 60°C with shaking at 500 rpm. All experiments were conducted in triplicates and uncertainties represent standard deviations.

Compound	Removal by pLac_*Gy*_ in absence of ABTS (%)	Removal by pLac_*Gy*_ in presence of ABTS (%)
Guaiacol	19 ± 1.5	93 ± 0.2
Phenol	14 ± 1.9	55 ± 1.9
BPA	8 ± 0.8	25 ± 0.7

## Conclusion

There are significant advances in the development and application of enzyme technology for pollutant degradation in recent years. In particular, several pilot scale studies of laccase-based wastewater treatments of persistent and emerging organic contaminants (which contain a significant amount of aromatic organic compounds) are now being trialled with promising results for pollutant degradation under realistic operational conditions (Gasser et al., 2014). Despite these advances, the number of laccases available for commercial or industrial-scale operations was still limited especially when compared to the range of catalysts that are available for chemocatalysis. Therefore, it is imperative that we continue to broaden the range of laccases that could be used for different operations (e.g. by identifying novel laccases), as well as improving further downstream parameters through methods such as immobilisation and protein engineering.

In this work, we turned to extremophiles for the search of new laccases. Extremophiles are organisms that have evolved to withstand “extreme” environmental conditions that most mesophilic organisms are unable to survive in. These organisms produce stable proteins that are adapted to function in unusual conditions and therefore are a good source of enzyme biocatalysts. Our work uncovered a putative laccase from a thermophilic bacterium, *G. yumthangensis*. pLac_*Gy*_ exhibited stability over a wide range of pH and was functional at elevated temperatures. In addition, pLac_*Gy*_ also showed high tolerance towards the presence of salt. By using organic dyes and phenolic pollutants as model compounds, we found that pLac_*Gy*_ could facilitate their degradation by using ABTS as a redox mediator. In a recent review, Arregui et al. produced a comparative table (Table 3 in Arregui et al., 2019) highlighting the production, reaction condition and results of a selection of bacterial laccases. Supplementary Table S2 is based on this table with additional rows for pLac_*Gy*_. Although it is difficult to compare the kinetic parameters between these reported laccases (as they were conducted under different conditions), pLac_*Gy*_ has comparable k_cat_/K_m_ value (64.6 s^−1^ M^−1^) as a recombinant laccase from *Klebsiella pneumoniae* (193 s^−1^ M^−1^) (Liu et al., 2017). However, both laccases have inferior k_cat_/K_m_ values than a recombinant laccase from *Bacillus pumilus* (160,000 s^−1^ M^−1^) (Luo et al., 2018) as well as Lac1326 (164,000 s^−1^ M^−1^) (Yang et al., 2018) owing to their low K_m_ values for ABTS (0.25 and 0.21 mM respectively) when compared to pLac_*Gy*_. Finally, it is important to note that, in order to progress pLac_*Gy*_ to become a biocatalyst that could be applied industrially, further optimisation is needed to expand its stability and substrate specificity. In addition, toxicity studies should also be conducted to evaluate the toxicity of the optimised pLac_*Gy*_ biocatalyst, the organic pollutants that the optimised pLac_*Gy*_ biocatalyst is employed to degrade, and the degradation products. Nonetheless, our work has led to the characterisation of a novel laccase and our results serve as a proof-of-principle study for the potential application of pLac_*Gy*_ for the biotreatment of wastewater to degrade aromatic organic compounds.

## Data Availability

The original contributions presented in the study are included in the article/Supplementary Material, further inquiries can be directed to the corresponding author.
