# Supplementary material for: Novel Thermophilic Bacterial Laccase for the Degradation of Aromatic Organic Pollutants
Source: Front Chem. 2021 Oct 20;9:711345. doi: 10.3389/fchem.2021.711345 (PMC8564365; doi:10.3389/fchem.2021.711345)
Supplement: Supplementary file 1 [file DataSheet1.pdf]

Supplementary Material

Novel thermophilic bacterial laccase for the degradation of aromatic organic pollutants

Nabangshu Sharma and Ivanhoe K.H. Leung

1 Supplementary Figures and Table

1.1 Supplementary Figures

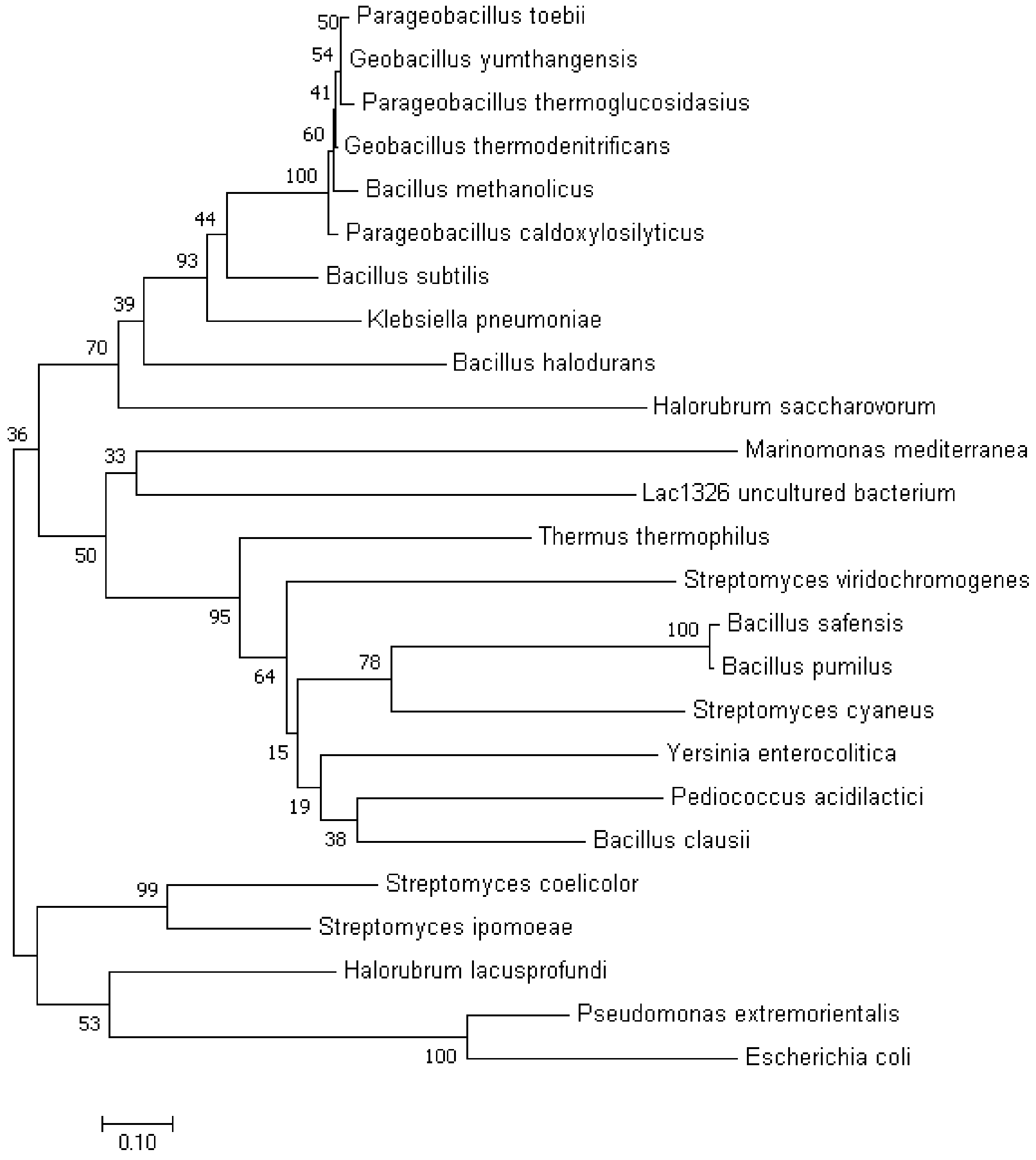

Figure S1: Phylogenetic analysis of $pLac_{Gy}$ with 24 other bacterial laccases. The amino acid sequences were obtained from NCBI database and were aligned using MEGA 7.0. The phylogenetic tree was constructed using the neighbour-joining method. Bootstrap values were from 100 replicates.

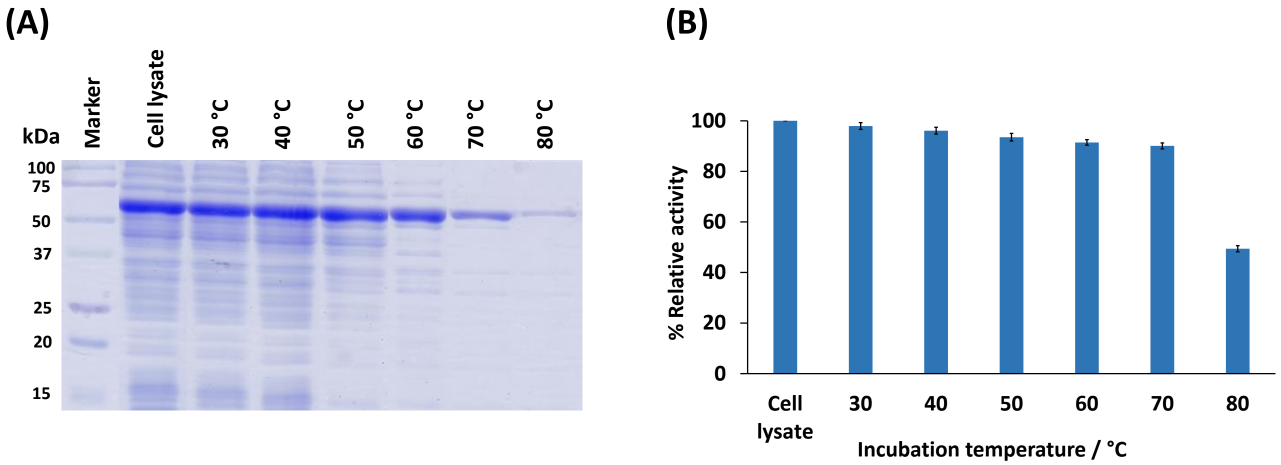

Figure S2: (a) SDS-PAGE analysis of pLac$_{Gy}$ purified by the heat precipitation purification method. Lane 1: protein ladder with the corresponding molecular weights on the left hand side of the gel; Lane 2: *E. coli* cell lysate overexpressed with pLac$_{Gy}$; Lanes 3-8: Purity of pLac$_{Gy}$ after heat precipitation treatment at different temperatures. The intense band at ~60 kDa corresponds to the recombininantly produced pLac$_{Gy}$, which was expressed with a N-terminal polyhistidine tag and thrombin cleavage site; (b) Laccase activity (as monitored by the oxidation of ABTS) of *E. coli* cell lysate overexpressed with pLac$_{Gy}$ with and without heat precipitation treatments. Reaction mixture included 1 µL of the cell lysate sample, 1 mM ABTS, 50 µM $CuCl_2$ in 0.04 M Britton-Robinson buffer (pH 5.0). Reaction temperature was 60 ºC and incubation time was 20 minutes. All experiments were conducted in triplicates and error bars represent standard deviations.

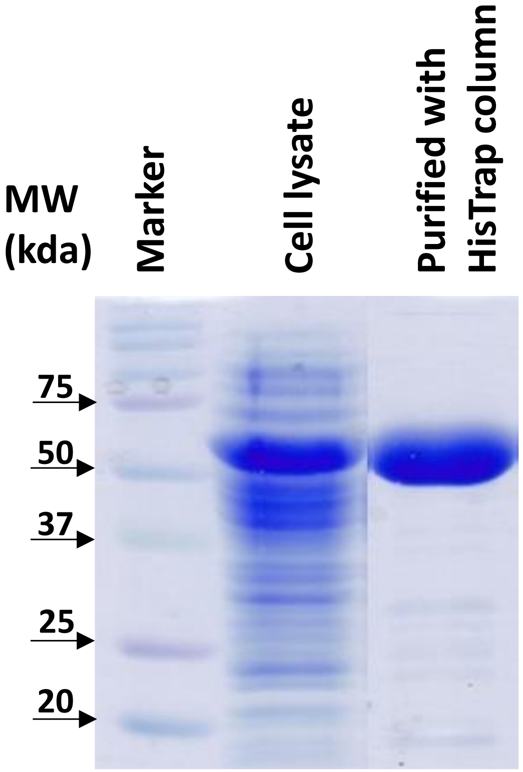

Figure S3: SDS-PAGE analysis of pLac$_{Gy}$ purified by IMAC. Lane 1: protein ladder with the corresponding molecular weights on the left hand side of the gel; Lane 2: *E. coli* cell lysate overexpressed with pLac$_{Gy}$; Lanes 3: Purity of pLac$_{Gy}$ after elution. The intense band at ~60 kDa corresponds to the recombininantly produced pLac$_{Gy}$, which was expressed with a N-terminal polyhistidine tag and thrombin cleavage site.

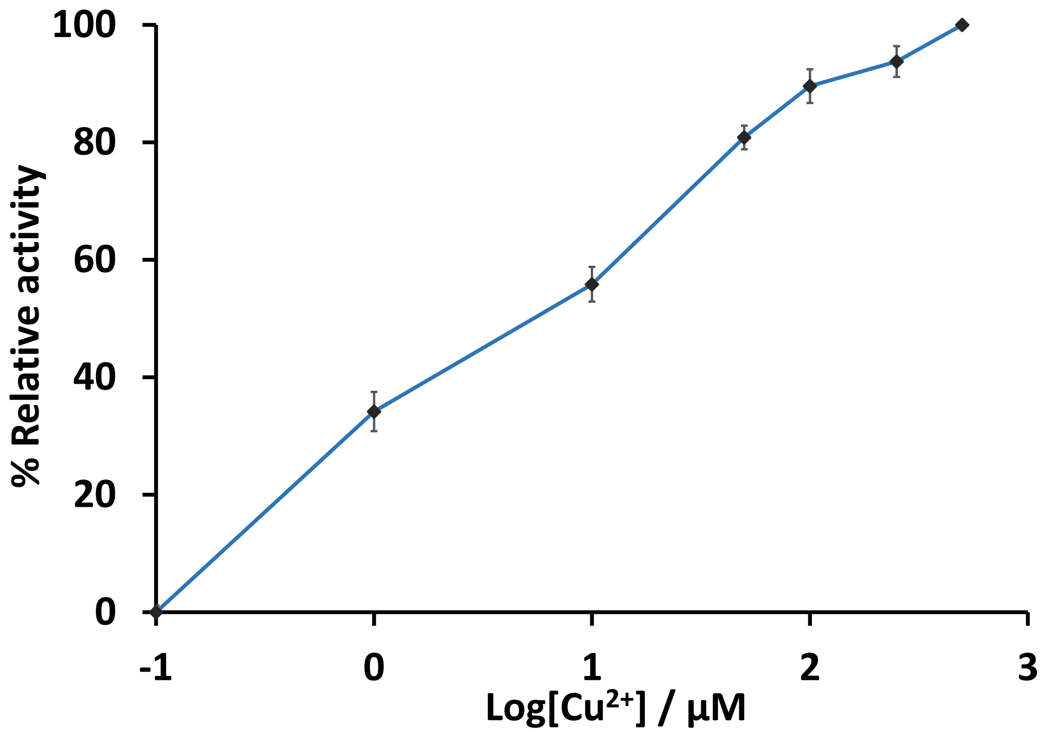

Figure S4: Effect of Cu^{2+} supplementation on the catalytic activity of the IMAC-purified pLac$_{Gy}$. Assay temperature was 60 ºC. Assay mixture included 0.5 µM pLac$_{Gy}$, 1 mM ABTS, varying concentration of $CuCl_2$ in 0.04 M Britton-Robinson buffer (pH 5.0). The relative activity at 500 µM Cu^{2+} concentration was set at 100%. All experiments were conducted in triplicates and error bars represent standard deviations. Lines were drawn between data points to aid visualisation.

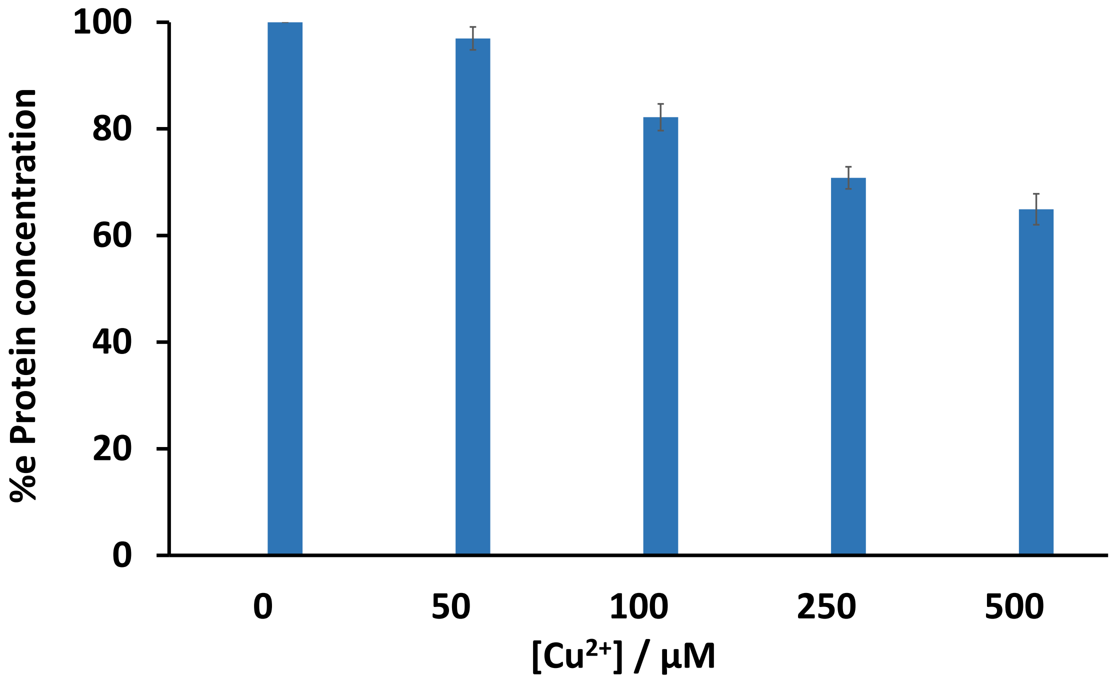

Figure S5: Effect of Cu^{2+} supplementation on the stability of the IMAC-purified pLac$_{Gy}$. Residual protein concentration was measured using the Bradford assay after pre-incubating the enzyme with different Cu^{2+} concentrations in 0.04 M Britton-Robinson buffer (pH 5.0) for 6 hours. The concentration of the enzyme without pre-incubation was set at 100%. All experiments were conducted in triplicates and error bars represent standard deviations.

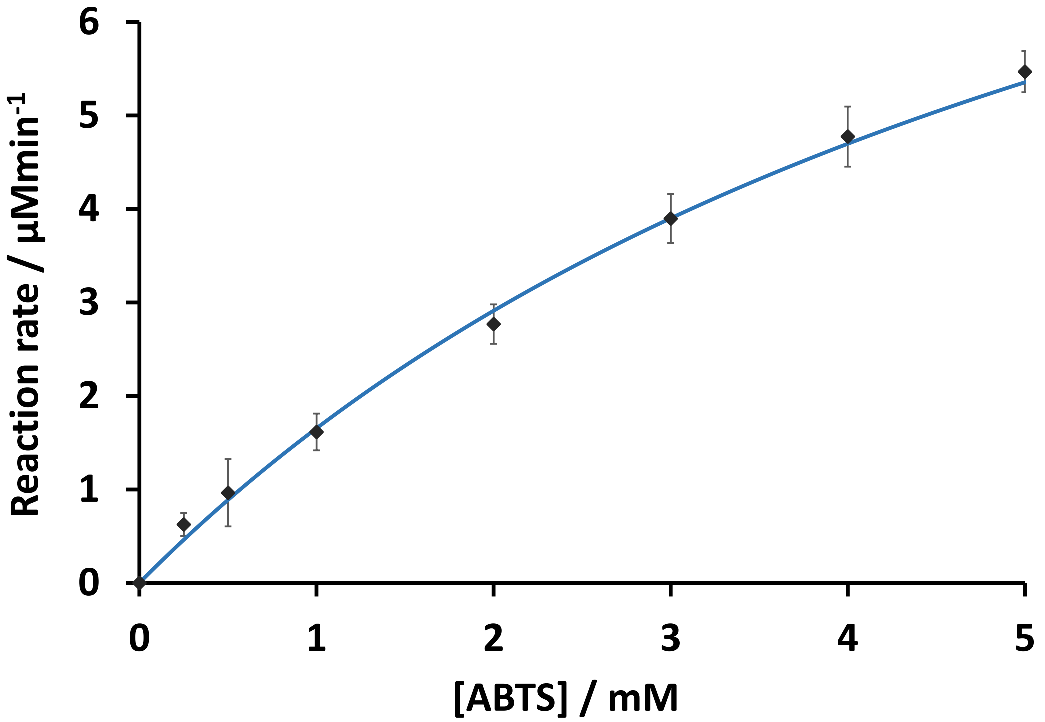

Figure S6: A plot of the initial rates of pLac$_{Gy}$-catalysed oxidation reaction of ABTS versus ABTS concentration. Assay mixture included 0.5 µM pLac$_{Gy}$, varying concentrations of ABTS, 50 µM $CuCl_2$ in 0.04 M Britton-Robinson buffer (pH 5.0). Reaction temperature was 60 ºC and incubation time was 5 minutes. The curve was fitted using the Michaelis-Menten reaction kinetics equation to determine kinetic parameters. All experiments were conducted in triplicates and error bars represent standard deviations.

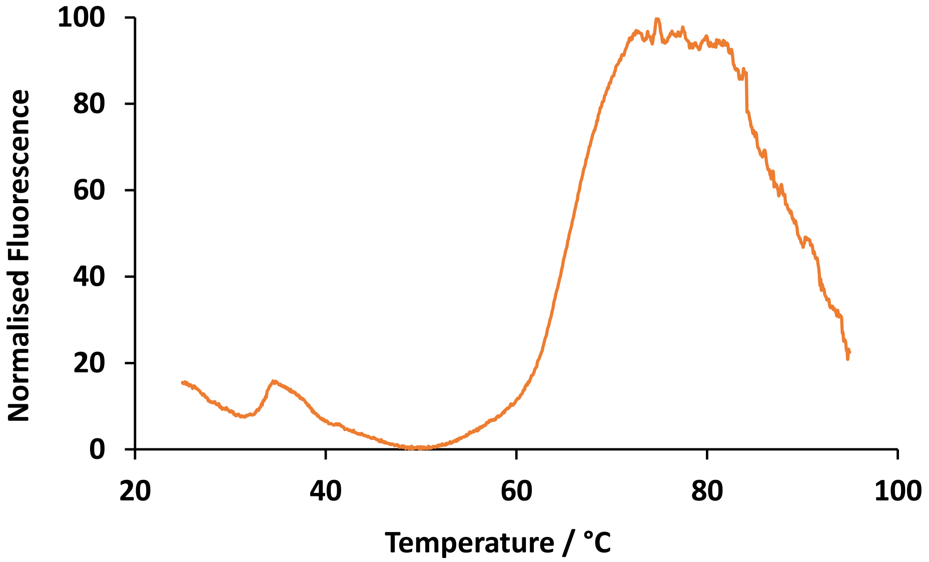

Figure S7: Protein melt curves showing the denaturing temperature (T_m) of pLac$_{Gy}$. The T_m for pLac$_{Gy}$ was found to be 65 °C. Data were collected at 1 °C intervals from 25 °C through 95 °C. The fluorescence dye used is SYPRO Orange.

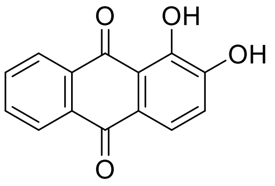

Alizarin

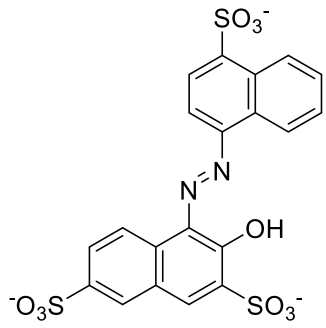

Acid red 27

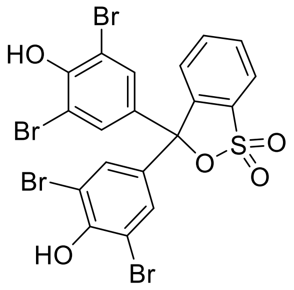

Bromophenol blue

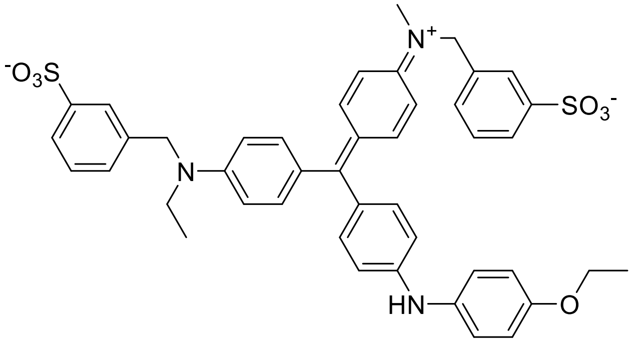

Coomassie brilliant blue

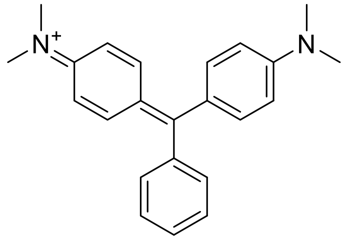

Malachite green

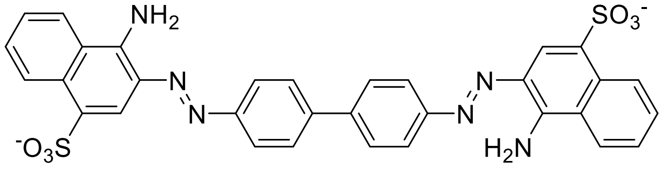

Congo red

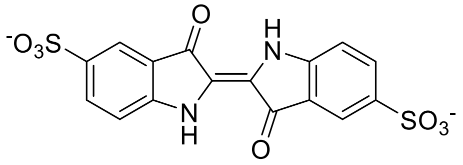

Indigo carmine

Figure S8: Structures of the seven organic dyes used in this study.

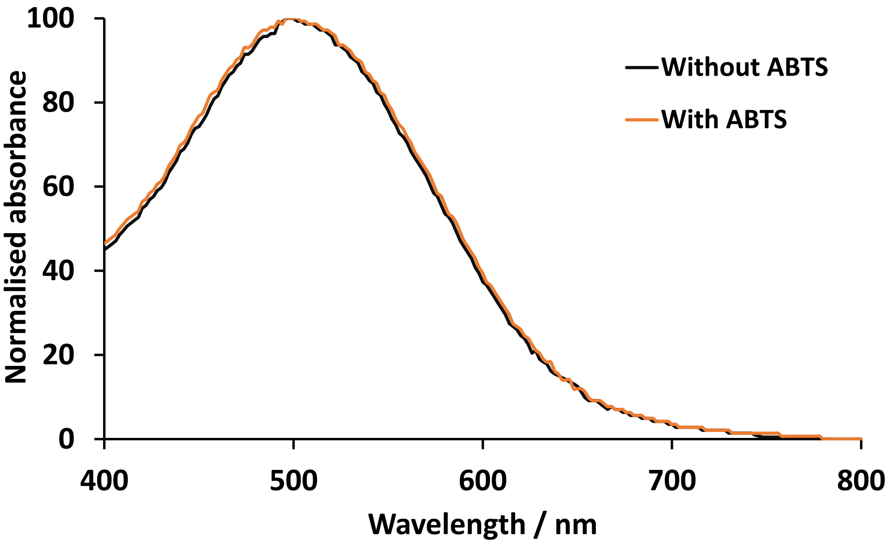

Figure S9: The absorbance spectrum of alizarin (0.05 mg/mL) in absence (black) and presence (orange) of 1 mM ABTS in Britton-Robinson buffer (pH 5.0). Maximum absorbance of the dye occurs at a wavelength of 496 nm.

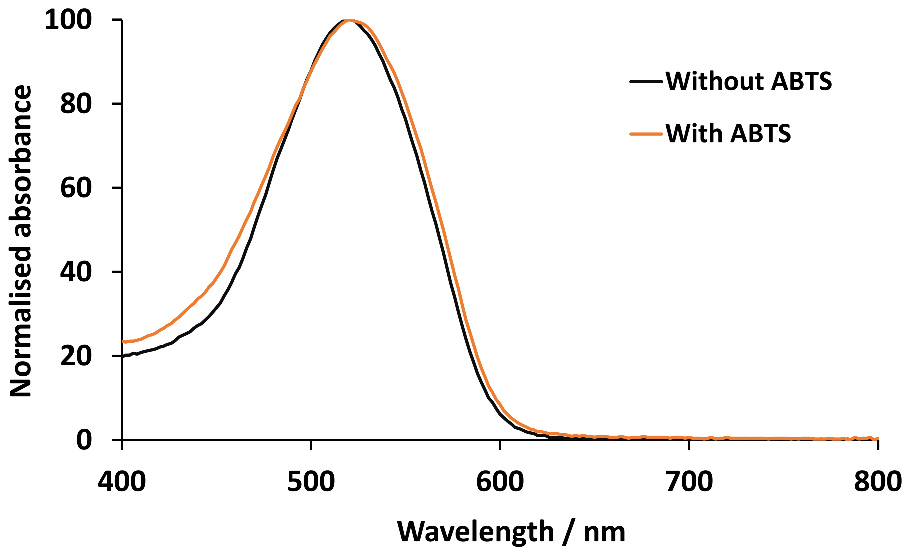

Figure S10: The absorbance spectrum of acid red 27 (0.05 mg/mL) in absence (black) and presence (orange) of 1 mM ABTS in Britton-Robinson buffer (pH 5.0). Maximum absorbance of the dye occurs at a wavelength of 520 nm.

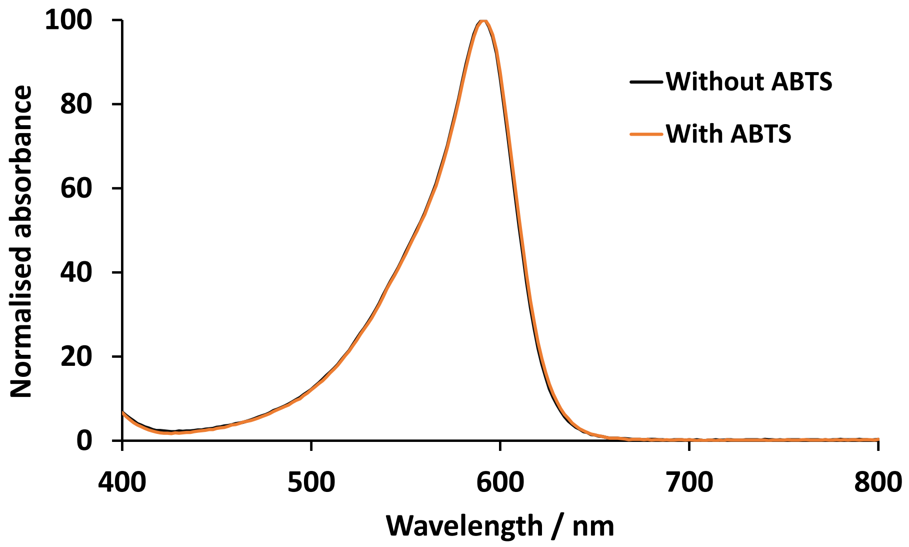

Figure S11: The absorbance spectrum of bromophenol blue (0.05 mg/mL) in absence (black) and presence (orange) of 1 mM ABTS in Britton-Robinson buffer (pH 5.0). Maximum absorbance of the dye occurs at a wavelength of 592 nm.

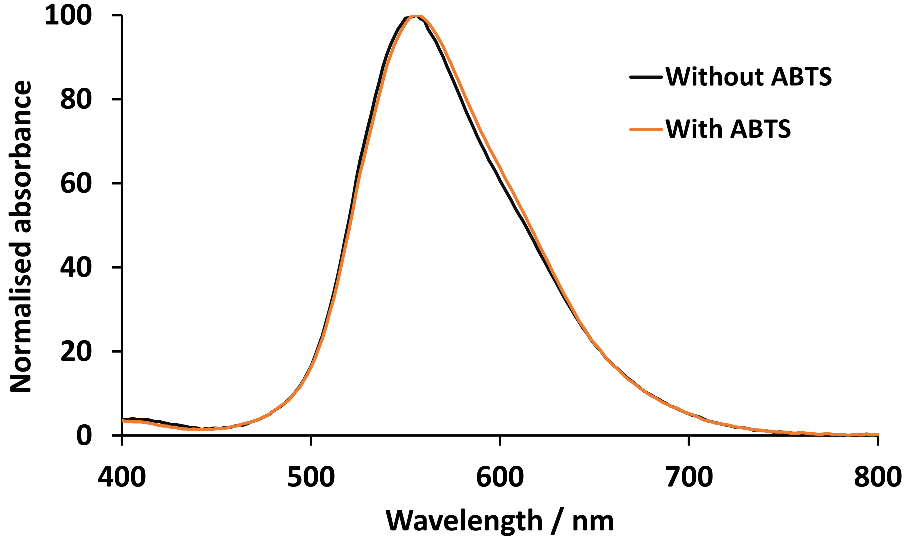

Figure S12: The absorbance spectrum of Coomassie brilliant blue R-250 (0.05 mg/mL) in absence (black) and presence (orange) of 1 mM ABTS in Britton-Robinson buffer (pH 5.0). Maximum absorbance of the dye occurs at a wavelength of 556 nm.

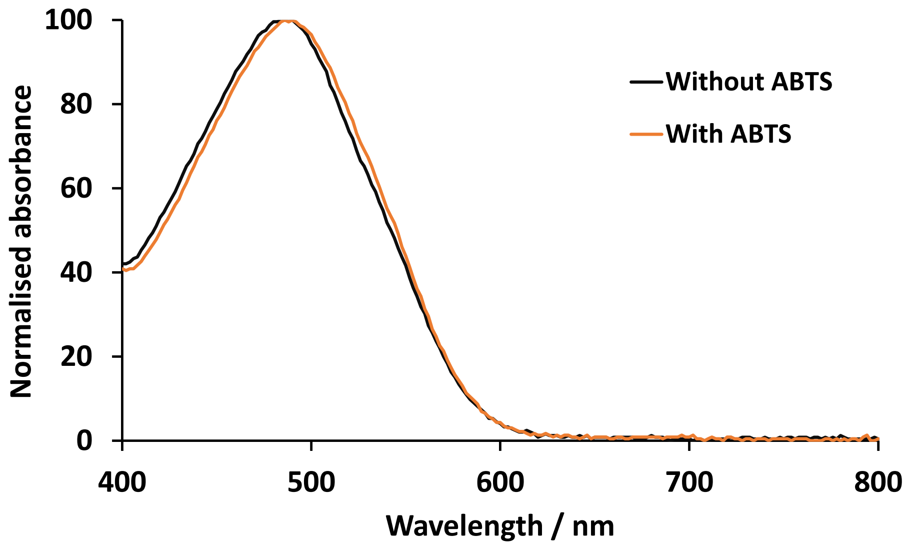

Figure S13: The absorbance spectrum of Congo red (0.05 mg/mL) in absence (black) and presence (orange) of 1 mM ABTS in Britton-Robinson buffer (pH 5.0). Maximum absorbance of the dye occurs at a wavelength of 490 nm.

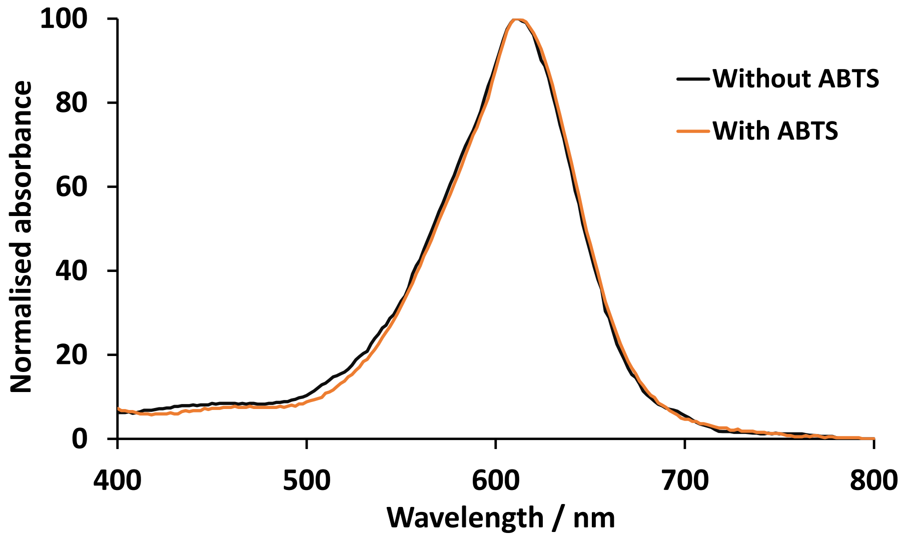

Figure S14: The absorbance spectrum of indigo carmine (0.05 mg/mL) in absence (black) and presence (orange) of 1 mM ABTS in Britton-Robinson buffer (pH 5.0). Maximum absorbance of the dye occurs at a wavelength of 610 nm.

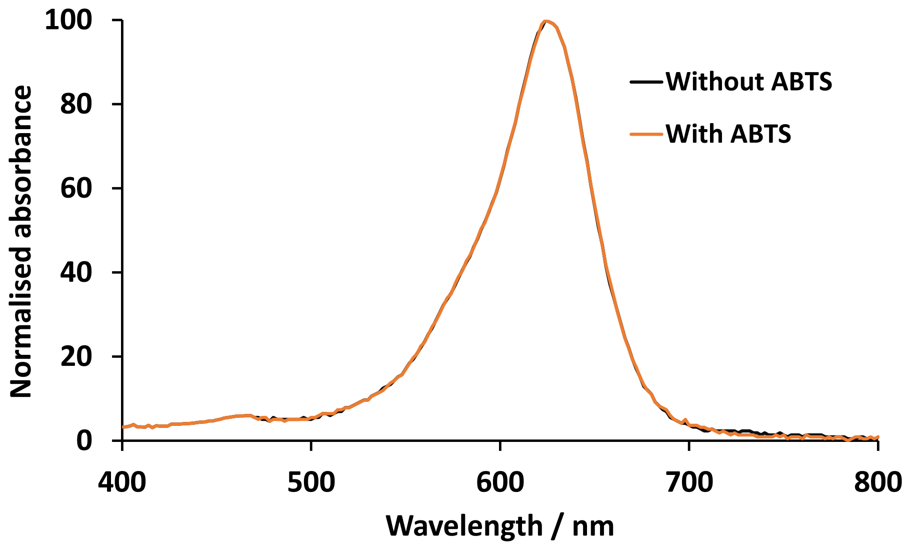

Figure S15: The absorbance spectrum of malachite green (0.05 mg/mL) in absence (black) and presence (orange) of 1 mM ABTS in Britton-Robinson buffer (pH 5.0). Maximum absorbance of the dye occurs at a wavelength of 624 nm.

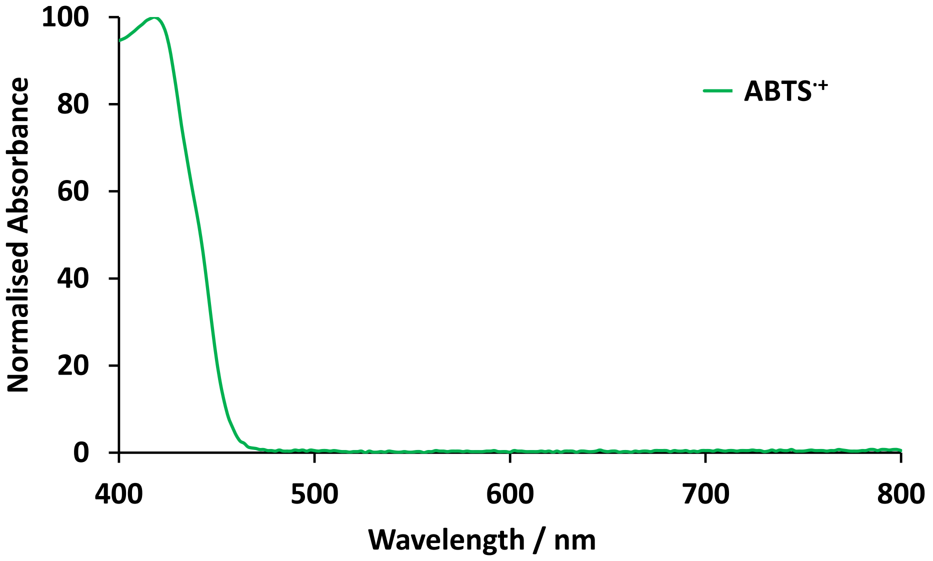

Figure S16: The absorbance spectrum of the oxidised product of 1 mM ABTS ($ABTS^{\cdot+}$) in Britton-Robinson buffer (pH 5.0). Maximum absorbance occurs at a wavelength of 420 nm.

1.2 Supplementary Table

ATGAAGAAGCTGCTGGTTGGCACCATTCTGGCGGGCGTGGTTGCGATTGGTGCGGCGTGCAGCAACAACGCGAGC

CACAGCAGCATGCAGGGCCACGATATGAGCAACATGAACATGAAAGAGGAGAACACCACCAAGGACAGCAGCAAA

CAACTGCCGCTGGCGATCAACACCGAGGTTCTGAGCGGCAAGGAAATTAACCTGACCGCGAAAGAGGCGCTGCTG

CAGATCAACGATAAGGTGAAACTGCCGGTTTACACCTATAACGGTAGCGTGCCGGGCGCGCAGATCCGTATTAAG

CAAGGCGACCGTGTTAAGATCAACTTCAAAAACGAGCTGCCGGAACCGACCACCATTCACTGGCACGGTTACCCG

GTGCCGAACAGCCAAGATGGTGTGCCGGGCGTTACCATGAACGCGATCAAACCGGGCGAGACCTTCACCTATGAA

TTTACCGCGACCGTTCCGGGCACCTACTTTTATCACAGCCACCAGGAGAGCGCGAAGCAAGTGGACAAAGGTCTG

TACGGCACCCTGATTGTTGAACCGAAGAACGAGGAAAAAGTGGACCGTGATTATACCCTGGTTCTGGATGAGTGG

ATGAGCAACCCGGACGAAGGCAACATGCACATGAGCGGTATGGACCACAGCAACATGGGTCACGGCAACAGCAGC

GACAACCAGCACATGGATATGAGCAACATGGGCCACGACATGAGCATGTACGACATCTTCACCATTAACGGCAAG

AGCGGCAGCGCGGTGAAGCCGCTGAAAGTTAAGAAAGGTGAAAAAGTGCGTCTGCGTCTGGTTAACGCGGGCTAT

ATGAGCCACAAGCTGCACCTGCACGGTCACGAGTTCAAAATCGTTGCGACCGATGGTCAGCTGCTGAAGGACCCG

CAGCCGATCAAAGACGAACTGCTGAACATTGCGCCGGGCGAGCGTTACGACATCGAATTTATTGCGAACAACCCG

GGTGAATGGCTGCTGGAATGCCACGGTGACATGGAGGGCACCGATGGCATGAAGGTTAAAATCCAGTACGAAGGT

CAAACCAACAACACCGACAAGGCGAACGCGAAAGAGGACCTGCCGATTGTGGATATGACCAAGTATGGCAAACAC

GAGCTGGGCCAGTTCACCCTGGACCAAAAATACGATGTTGAATATACGATGGACCTGGGCACCGCGATGGGTAAA

GACGGCACCATCTTTACCATTAACGGCAAAACCTACCCGGAAACCGCGCCGGTGAACGTTAAGAAAGGTGATCTG

GTGAAGGTTAAACTGGTGAACAACAGCCCGAAGGACCTGCACCCGATGCACCTGCACGGTCACTTCTTTCAGGTT

CTGAGCAAGAACGGCAAACCGATCACCGGTAGCCCGCTGATTAAGGATACCCTGAACCTGAAACCGGGCGAGGAA

TACGTGGTTGCGTTCAAAGCGGATAACCCGGGCAACTGGATGTTTCATTGCCATGACCTGCACCATGCGAGCGCG

GGTATGGTGACCGAGGTTAAGTACAAGGACTACAAGAGCGATTACACCCCGAACCCGAACGACACCACCAACAAA

CCGGAATAA

Table S1: Codon optimised sequence encoding pLac$_{Gy}$ used in this study.

Laccase Source	Applied enzyme form	Type of culture, ingredients	Application	Reaction parameters	Results obtained	Main putative mechanisms involved	Enzyme kinetics	Working pH range	Working temperature range
Pharmaceutical compounds									
Recombinant laccase from *Yersinia enterocolitica* expressed in *E. coli* (Singh et al., 2016)	CS	Expressed in *E. coli* with IPTG induction	Degradation of non-steroidal anti-inflammatory drugs	*E. coli* cells harboring laccase from *Y. enterocolitica* were treated with 0.1 mM Tween 80 and $CuCl_2$ 0.2 mM in buffer pH 6, at 45 °C for 30 min. After that, diclofenac and aspirin were added at 5 mg/L incubated at 45 °C	After 24 h, both diclofenac and aspirin were fully degraded	In the case of the diclofenac, laccase oxidation by hydroxylations of 4′ or 5′ positions of the second benzene ring could be the modifications	K_m and V_{max} values of 0.68 mM and 125 µM min^{-1} respectively for ABTS as a substrate	Optimum pH of 3.0 with ABTS as a substrate.	Optimal working temperature of 30 °C for ABTS.
Streptomyces cyaneus (Margot et al., 2013)	C	Production of laccase was done in ISP9 mineral medium, with soy flour (10 g/L) as carbon source and a copper concentration of 1 mg/L $CuSO_4 \cdot 5\ H_2O$. Cultures were incubated at 30 °C for 23 days. Cell-free culture supernatant was collected, filtered and stored as enzyme source	Degradation of non-steroidal anti-inflammatory drugs (diclofenac: DFC) and mefenamic acid: MFA)	The reactions were performed in citrate phosphate buffer (30–40 mM) at three different pH values (5, 6 and 7), with a pollutant concentration of 20 mg/L. 2000 U/L of crude enzyme preparation were used. The reactions were incubated in the dark at 25 °C for 12 days	The enzyme showed a high conversion rate under acidic conditions (pH 5, 6), with 50% of conversion after 2 days for DFC. With respect to MFA, the highest conversion was obtained in pH 6	Not discussed	Data not available	Active between pH 4.0-5.0 and stable between pH 5.0-9.0 with optimum operational pH of 4.5 for ABTS.	Active between 40-70 °C with optimum working temperature of 60 °C for ABTS.
Recombinant *Streptomyces ipomoea* SilA laccase expressed in *E. coli* (Mot et al., 2012)	F	2 L of LB medium at 37 °C were inoculated with 40 mL of an exponential-growth-phase culture. When exponential growth had resumed, the temperature was reduced to 28 °C, and SilA expression was induced with 1 mM IPTG. Purified enzyme was used	Degradation of fluoroquinolone antibiotics (ciprofloxacin: CIP, and norfloxacin)	The reactions were carried out in 50 mM phosphate buffer pH 8 at 35 °C, using 0.4 U/mL of laccase and 50 µg/mL of each fluoroquinolone. Several mediators at concentrations of 0.1, 0.3 and 0.5 mM were tested	After 24 h and with 0.5 mM acetosyringone, higher than 90% percent conversions were obtained for both antibiotics, with a detoxification effectiveness of 70% for CIP and 90% for norfloxacin	Possible oxidation of piperazine substituents	Data not available	Optimum working pH of 4.5 for ABTS as a substrate.	Stable between 0-50 °C.
Streptomyces mutabilis A17 (Reda et al., 2019)	F	The culture was done in a solid-state fermentation, using cotton seed cake (5 g/L) as substrate, supplemented with mineral salts and glucose 1% (w/v). The medium was inoculated with a spore suspension and incubated for 6 days at 35 °C. The laccase was extracted and purified	Degradation of sulfa antibiotics (sulfadiazine and sulfathiazole)	In a 100 mM citrate–phosphate buffer pH 6 were dissolved each sulfa drug, with a final concentration of 50 mg/L. To this solution were added the laccase (81.3 U/mg), and 1 mM HBT (mediator). The reaction was done at 50 °C for 60 min	Under the conditions previously described, 73 and 90% removal efficiencies were achieved to sulfadiazine and sulfamethoxazole solutions, respectively. Moreover, the reaction products showed less antibiotic effect in bacterial cultures	Not reported	Data not available	Active and stable between pH 7.0-9.0 for guaiacol as a substrate with optimum pH of 8.0.	Optimum working temperature of 40 °C with guaiacol as substrate.
Dye based pollutants									
Recombinant laccase from *Klebsiella pneumoniae* expressed in *E. coli* (Liu et al., 2017)	F	*E. coli* cells were grown in LB medium at 37 °C until 0.6–0.8 DO. After that, were induced with IPTG for 20 h at 16 °C. Purified laccase	Decolorization of synthetic dyes	The reactions were done with 0.025, 0.05 and 0.1 U of the purified enzyme, in 50 mM citrate–phosphate buffer (pH 4.0 and 7.5) and 15 µL dye solution (100 mg/L) at 70 °C in 90 min	All the 10 dyes tested were efficiently oxidized under by the enzyme alone in both acidic and neutral conditions	Not reported	K_m and k_{cat} values of 5.33 mM and 1.02 s^{-1} respectively with ABTS as a substrate	Active between pH 3.5-4.5 and stable between pH 4.0-9.0 with optimum working pH of 4.0 for ABTS as substrate.	Active and stable between 30-40 °C with optimum working temperature of 35 °C towards ABTS.
Recombinant *E. coli* K-12 CueO expressed in *Pichia pastoris* (Ma et al., 2017)	F	48 h culture in BMGY at 28 °C. The induction was made with methanol 1% and 0.2 mM CuSO4 for 144 h, feeding methanol each 24 h. Purified laccase	Decolorization of synthetic dyes	The reactions were carried out at 55 °C in phosphates buffer 50 mM, pH 7.5, with a dye concentration of 80 mg/L and supplemented with 1 mM CuSO4 and 0.1 mM of acetosyringone as mediator. 1 µL of purified laccase was used	After 3 h of reaction, the laccase decolorized almost all the Congo red and malachite green tested. After 24 h, 90% of the remazol brilliant blue R were degraded	Not mentioned	Data not available	Active between pH 2.0-3.5 with optimum working pH of 3.0 for ABTS as substrate.	Active between 45-65 °C with optimum working temperature of 55 °C towards ABTS.
Recombinant and mutant laccase WLF from *Bacillus pumilus* expressed in *E. coli* (Luo et al., 2018)	F	Culture grown at 37 °C in LB medium until 0.5 DO. After that, were added IPTG (0.4 mM) and $CuSO_4$ (0.25 mM) and maintained at 15 °C for 24 h. Purified laccase	Decolorization of synthetic dyes	Reaction mixture consisted in 0.25 mg of dye, 2 mg/L of purified laccase 1 mM de acetosyringone in 5 mL of 100 mM carbonate buffer pH 10, at 37 °C	Highest transformations of all the dyes tested. The efficiency with aromatic heterocyclic dyes was lower compared with azo, anthraquinonic and triphenylmethane dyes	Not reported	K_m and k_{cat} values of 0.25 mM and 40.04 s^{-1} respectively with ABTS as a substrate	Active between pH 3.0-4.5 towards ABTS and stable between pH 3.0-12.0 with optimum working pH of 3.5.	Active between 40-90 °C and stable upto 50 °C with optimum working temperature of 50 °C towards ABTS.
Bacillus safensis S31 (Siroosi et al., 2018)	SS	*B. saensis* cells were cultured on nutrient agar sporulation medium and incubated at 35 °C for 4 days. After that incubation time, spore suspension was prepared, used as a source of laccase	Decolorization of synthetic dyes (malachite green, toluidine blue and reactive black 5)	To 2 mL of 50 mM acetate buffer (for pH values of 3–6) or 50 mM Tris buffer (for pH values of 7 and 8) were added the spore laccase suspension (8 U/L) and dye (final concentration of 10 mg/L). The effect of ABTS (15 µM) as mediator was also studied. The reactions were carried out at 30 °C for 2 h	Almost all the oxidation conditions showed better results with ABTS. The highest decolorization values for malachite green and toluidine blue were achieved between 5 and 7 pH values, while with reactive black were between pH 3 and 5	Not mentioned	Data not available	Active between pH 4.0-6.0 with maximum activity at pH 5.0 towards ABTS.	Active between 25-40 °C with optimum activity at 30 °C towards ABTS.

Recombinant *Thermus thermophilus* SG0.5JP17-16 expressed in *Pichia pastoris* (Liu et al., 2015)	F	An inoculum of *Pichia pastoris* cells growth in BMGY medium were used to inoculate BMMY medium containing 0.1 mM $CuSO_4$. The culture was cultivated at 30 °C for 7 days with daily addition of 1% methanol. The enzyme was purified from the supernatant	Decolorization of synthetic dyes (reactive black B, reactive black WNN, congo red and remazol brilliant blue R)	A reaction mixture of 50 mM phosphates buffer pH 7.5, 10 µM $CuSO_4$, 50 mg/L dye and 40 U/L of purified laccase were heated at 70 °C for 24 h	After 24 h the decolorization efficiency for congo red, reactive black B and reactive black WNN was higher than 90%, while for remazol brilliant blue R was around 70%	Not mentioned	Data not available	Active between pH 4.0-6.0 towards ABTS and stable between pH 4.0-11.0 with optimum working pH of 4.5.	Active between 60-100 °C with optimum working temperature of 90 °C using guaiacol as a substrate.
Recombinant *Streptomyces ipomoeae* SilA, expressed in *E. coli* (Blánquez et al., 2016)	F	*E. coli* BL21 (DE3) transformed and containing the codifying gene of SilA	Decolorization of synthetic dyes	Laccase SilA and three mediators (0.1 mM), acetosyringone (AS), syringaldehyde (SA) and methyl syringate (MeS), by 24 h at 35 °C pH 8, and different dyes (acid black 48: AB48, acid orange 63: AO63, reactive black 5: RB5, orange II: OII, tartrazine: TART, azure B: AB, indigo carmine: IC, cresol red: CR	Laccase and mediators such as AS and MeS enhanced the decolorization and detoxification of a variety of textile dyes, principally RB5, OII, and IC, diminishing the toxicity of acid orange 63, tartrazine	The oxidation of MeS (which has the weakest acceptor group at the para-position) gives an stable phenoxy radical	Data not available	Optimum working pH of 4.5 for ABTS as a substrate.	Stable between 0-50 °C.
Recombinant *Geobacillus yumthangensis* laccase expressed in *E. coli* (This work)	F	*E. coli* BL21 (DE3) transformed with pET-28a(+) plasmid encoding full length pLac$_{Gy}$. Culture grown in 2YT media supplemented with 0.25 mM $CuCl_2$ at 37 °C until optical density of 0.6. Protein expression was induced with 0.1 mM IPTG (final concentration) followed by incubation at 18 °C for 16 h. Purified laccase	Decolorization of synthetic dyes	Reaction mixture included 1 µM pLac$_{Gy}$, 1 mM ABTS, 50 µM $CuCl_2$, 0.05 mg/mL organic dye in Britton-Robinson buffer (pH 5.0). The mixture was incubated overnight at 60 ºC with shaking at 500 rpm. Seven different dyes were tested: alizarin, acid red 27, Congo red, bromophenol blue, Coomassie brilliant blue R-250, malachite green and indigo carmine.	Effective decolourization (>70%) of five (out of the seven) tested dyes in the presence of ABTS as mediator. These include alizarin, Congo red, Coomassie brilliant blue R-250, malachite green and indigo carmine.	Not studied	K_m, V_{max} and k_{cat} values of 6.35 mM, 12.16 µM min^{-1} and 0.41 s^{-1} respectively with ABTS as a substrate	Active between pH 5.0-6.0 towards ABTS and stable between pH 3.0-12.0 with optimum working pH of 5.0.	Active between 50-80 °C and stable between 0-60 °C with optimum working temperature of 60 °C towards ABTS.
Plastic and polycyclic aromatic hydrocarbons (PAHs) compounds									
Recombinant *B. subtilis* CotA expressed in *E. coli* (Zeng et al., 2016)	F	*E. coli* cells harboring the plasmid with the CotA gen were grown at 37 °C in LB medium. When the culture reaches 0.6 DO, were added IPTG and $CuSO_4$ to final concentrations of 0.1 and 0.25 mM, respectively. The incubation temperature was reduced to 25 °C for 6 h. After that, the culture agitation was stopped for 12 h. Purified laccase was used	Degradation of PAHs (anthracene, pyrene benzo[α]pyrene, phenanthrene, fluoranthene, etc.)	The reactions were carried out in 50 mM acetate buffer pH 4 with 10% acetonitrile, with PAHs concentrations from 0.1 to 1 mg/L and laccase concentration of 3 U/mL. The reactions were incubated for 24 h at 20, 40 and 60 °C	Just anthracene and benzo[α]pyrene were significantly oxidized (almost complete oxidations at 60 °C), the other ones had degradation values from 0 to 40% in all the conditions tested	Not reported	Data not available	Active between pH 4.0-6.0 towards ABTS with optimum working pH of 4.0.	Active between 50-80 °C and stable at 60 °C for 4h with optimum working temperature of 60 °C towards ABTS.
Streptomyces cyaneus (Margot et al., 2013)	C	Production of laccase was done in ISP9 mineral medium, with soy flour (10 g/L) as carbon source and a copper concentration of 1 mg/L $CuSO_4 \cdot 5H_2O$. Cultures were incubated at 30 °C for 23 days. Cell-free culture supernatant was collected, filtered and stored as enzyme source	Degradation of bisphenol A	The reactions were performed in citrate phosphate buffer (30–40 mM) at three different pH values (5, 6 and 7), with a pollutant concentration of 20 mg/L. 2000 U/L of crude enzyme preparation were used. The reactions were incubated in the dark at 25 °C for 12 days	Under all the conditions tested after 2 days there was full degradation, especially at pH 5 and 6	Not reported	Data not available	Active between pH 4.0-5.0 and stable between pH 5.0-9.0 with optimum operational pH of 4.5 for ABTS.	Active between 40-70 °C with optimum working temperature of 60 °C for ABTS.
Recombinant *G. yumthangensis* laccase expressed in *E. coli* (This work)	F	See above	Removal of bisphenol A, phenol and guaiacol	Reaction mixture included 1 µM pLac$_{Gy}$, 1 mM ABTS, 50 µM $CuCl_2$, 0.5 mM phenol, guaiacol or bisphenol A, in Britton-Robinson buffer (pH 5.0). The mixture was incubated overnight at 60 ºC with shaking at 500 rpm.	In the presence of ABTS as mediator, removal of guaiacol by >90%, phenol by >50% and bisphenol A by ~25% after overnight.	Not studied	See above	See above	See above

Table S2: Comparative table of selected bacterial laccases highlight their production, kinetics and their application in the degradation of pharmaceuticals, organic dyes, and plastic and polycyclic aromatic hydrocarbons. This table was adapted from Arregui et al., 2019, with extra rows added for pLac$_{Gy}$. Key: CS cell suspension, SS spore suspension, F free purified enzyme, C crude enzyme extract, IPTG isopropyl β-D-1-thiogalactopyranoside, DFC diclofenac, MFA mefenamic acid, AzBTS-(NH4)2 2,2′-azino-bis(3-ethylbenzothiazoline-6-sulfonic acid) diammonium salt, ABTS diammonium 2,2′-azino-bis(3-ethylbenzothiazoline-6-sulfonate), PAHs polycyclic aromatic hydrocarbons, BMGY buffered glycerol-complex medium, CIP ciprofloxacin, buffered methanol-complex medium

2 References

Arregui L, Ayala M, Gómez-Gil X, Gutiérrez-Soto G, Hernández-Luna CE, Herrera de los Santos M, Levin L, Rojo-Domínguez A, Romero-Martínez D, . Saparrat MCN, Trujillo-Roldán MA, Valdez-Cruz NA. Laccases: structure, function, and potential application in water bioremediation. Microb Cell Factories 2019;18:200.

Blánquez A, Guillén F, Rodríguez J, Arias ME, Hernández M. The degradation of two fluoroquinolone based antimicrobials by SilA, an alkaline laccase from *Streptomyces ipomoeae*. World J Microbiol Biotechnol. 2016;32(3):52.

Liu H, Cheng Y, Du B, Tong C, Liang S, Han S, Zheng S, Lin Y. Overexpression of a novel thermostable and chloride-tolerant laccase from *Thermus thermophilus* SG0.5JP17-16 in Pichia pastoris and its application in synthetic dye decolorization. PLoS ONE. 2015;10:1–14.

Liu Y, Huang L, Guo W, Jia L, Fu Y, Gui S, Lu F. Cloning, expression, and characterization of a thermostable and pH-stable laccase from *Klebsiella pneumoniae* and its application to dye decolorization. Process Biochem. 2017;53:125–34.

Luo Q, Wang KQ, Liao XR, Guan ZB, Chen Y, Cai YJ, Xia J. Functional expression enhancement of *Bacillus pumilus* CotA-laccase mutant WLF through site-directed mutagenesis. Enzyme Microb Technol. 2018;109:11–9.

Ma X, Liu L, Li Q, Liu Y, Yi L, Ma L, Zhai C. High-level expression of a bacterial laccase, CueO from *Escherichia coli* K12 in *Pichia pastoris* GS115 and its application on the decolorization of synthetic dyes. Enzyme Microb Technol. 2017;103:34–41.

Margot J, Bennati-Granier C, Maillard J, Blánquez P, Barry DA, Holliger C. Bacterial versus fungal laccase: potential for micropollutant degradation. AMB Express. 2013;3(1):63.

Mot A, Silaghi-Dumitrescu R. Laccases: complex architectures for one-electron oxidations. Biochemistry. 2012;77:1395–407.

Reda FM, El-Mekkawy RM, Hassan NS. Detoxification and bioremediation of sulfa drugs and synthetic dyes by *Streptomyces mutabilis* A17 laccase produced in solid state fermentation. J Pure Appl Microbiol. 2019;13(1):85–96.

Singh D, Rawat S, Waseem M, Gupta S, Lynn A, Nitin M, Ramchiary N, Sharma KK. Molecular modeling and simulation studies of recombinant laccase from *Yersinia enterocolitica* suggests significant role in the biotransformation of non-steroidal anti-inflammatory drugs. Biochem Biophys Res Commun. 2016;469:306–12.

Siroosi M, Amoozegar MA, Khajeh K, Dabirmanesh B. Decolorization of dyes by a novel sodium azide-resistant spore laccase from a halotolerant bacterium, *Bacillus safensis* sp. strain S31. Water Sci Technol. 2018;77(12):2867–75.

Zeng J, Zhu Q, Wu Y, Lin X. Oxidation of polycyclic aromatic hydrocarbons using *Bacillus subtilis* CotA with high laccase activity and copper independence. Chemosphere. 2016;148:1–7.
